# Pathway optimization by re-design of untranslated regions for L-tyrosine production in *Escherichia coli*

**DOI:** 10.1038/srep13853

**Published:** 2015-09-08

**Authors:** Seong Cheol Kim, Byung Eun Min, Hyun Gyu Hwang, Sang Woo Seo, Gyoo Yeol Jung

**Affiliations:** 1 School of Interdisciplinary Bioscience and Bioengineering, Pohang University of Science and Technology, 77 Cheongam-ro, Nam-gu, Pohang, Gyeongbuk 790-784, Korea; 2 Department of Chemical Engineering, Pohang University of Science and Technology, 77 Cheongam-ro, Nam-gu, Pohang, Gyeongbuk 790-784, Korea

## Abstract

L-tyrosine is a commercially important compound in the food, pharmaceutical, chemical, and cosmetic industries. Although several attempts have been made to improve L-tyrosine production, translation-level expression control and carbon flux rebalancing around phosphoenolpyruvate (PEP) node still remain to be achieved for optimizing the pathway. Here, we demonstrate pathway optimization by altering gene expression levels for L-tyrosine production in *Escherichia coli.* To optimize the L-tyrosine biosynthetic pathway, a synthetic constitutive promoter and a synthetic 5′-untranslated region (5′-UTR) were introduced for each gene of interest to allow for control at both transcription and translation levels. Carbon flux rebalancing was achieved by controlling the expression level of PEP synthetase using UTR Designer. The L-tyrosine productivity of the engineered *E. coli* strain was increased through pathway optimization resulting in 3.0 g/L of L-tyrosine titer, 0.0354 g L-tyrosine/h/g DCW of productivity, and 0.102 g L-tyrosine/g glucose yield. Thus, this work demonstrates that pathway optimization by 5′-UTR redesign is an effective strategy for the development of efficient L-tyrosine-producing bacteria.

L-Tyrosine is a commercially important compound, as it is widely used as a precursor of pharmaceutical drugs and other valuable chemicals such as flavonoids and alkaloids^1^,^2^,^3^,^4^,^5^. Traditionally, L-tyrosine was produced by extraction methods; however, the increasing demand for L-tyrosine is not adequately met by such methods due to low-yield production^6^,^7^. Microbial production is currently being spotlighted as a mean to efficiently produce L-tyrosine due to the feasibility of which has been enhanced in recent years by greater understanding of genetic information, the existence of various engineering tools, and simple scale-up processes^1^,^3^,^8^.

For several decades, many attempts have been made to improve L-tyrosine production using rational or combinatorial approaches. Such rational approaches, which include overexpression of pathway enzymes, introduction of feedback-resistant enzymes, and deregulation of regulators (e.g. TyrR protein) have allowed for improved L-tyrosine production by rationally increasing the carbon flux toward L-tyrosine production and removing the bottlenecks in the L-tyrosine biosynthetic pathway^9^,^10^,^11^,^12^,^13^. For example, Juminaga *et al.* overexpressed and modulated genes of the L-tyrosine biosynthetic pathway yielding 2.169 g/L of titer, 0.055 g/h/g DCW of productivity, and 0.438 g L-tyrosine/g glucose yield in flask culture^11^. Metabolic flux imbalance by indiscriminate deletion or overexpression of genes has, however, meant that these rational modifications to L-tyrosine production methods have not satisfactorily maximized L-tyrosine production. For this reason, precise control of gene expression levels or optimization of modular engineering may be required for metabolic pathway optimization. Combinatorial approaches for optimizing L-tyrosine production include the introduction of small RNA regulators, global transcriptional machinery engineering (gTME), and melanin-based high-throughput screening. Recently, Na *et al.* achieved 2.0 g/L of titer, 0.034 g/h/g DCW of productivity, and 0.1 g L-tyrosine/g glucose yield in flask culture using small RNA regulators^14^,^15^,^16^,^17^. Such approaches are advantageous to the production of large quantities of L-tyrosine because they overcome the limitations of rational approaches caused by a lack of understanding of complicated metabolic networks or pathways, and using such approaches, strain improvement is easily achieved via library screening. Despite these benefits, an efficient screening method and further optimization of metabolic pathways after screening are required in conjunction with combinatorial approaches in order to further enhance the efficiency of these methods.

The optimization of metabolic pathways for L-tyrosine production requires precise control of the expression levels of key enzymes at critical nodes, such as the phosphoenolpyruvate (PEP) node, which is both a precursor of the aromatic amino acid synthetic pathway and an intermediate of the glycolysis pathway^18^,^19^,^20^,^21^. If carbon flux at the phosphoenolpyruvate (PEP) node was increased toward the L-tyrosine biosynthetic pathway through genetic engineering, carbon flux to the tricarboxylic acid (TCA) cycle would be decreased. This flux decrease results in cell growth inhibition due to non-optimal overexpression, and the low cell density may in turn lead to low L-tyrosine production^22^,^23^. Thus, metabolic pathways should be optimized by precise control of the carbon flux between the L-tyrosine biosynthetic pathway and the TCA cycle at the PEP node for maximum L-tyrosine production.

Other researchers have previously reported on the regulation of expression levels by altering promoter strength or plasmid copy number; however, expression levels in these cases were not accurately regulated at a translational level^24^,^25^,^26^. The design tools such as RBS Calculator (v1.0) which calculate translation initiation rate have been developed to predict and to design 5′-UTR sequence for desired expression level^27^. It has been thoroughly tested and employed for optimizing various biological systems^28^,^29^,^30^,^31^. We have also developed UTR Designer after the RBS Calculator with conceptually similar free energy model, but differed in terms of investigating specific mRNA folding regions critical for ribosome accessibility by statistical approach using 456 5′-UTR variants in order to better capture the effect of structure changes in translation rate^32^. The UTR Designer was also proven to be useful designing short 5′-UTR sequences (25 base-pairs) to meet specific translation rate^33^,^34^,^35^,^36^,^37^,^38^,^39^. A recent study offered a molecular explanation showing how mRNA structures at upstream regions control translation rate and developed a new version of RBS Calculator (v2.0) capable of predicting and designing longer structured 5′-UTR sequences^40^. Combined engineering approaches which include promoter strength alteration and synthetic 5′-UTR sequence design may allow for the expression levels of specific genes to be precisely controlled and thus for the effective optimization of metabolic pathways.

In this study, an *Escherichia coli* strain was generated to produce large quantities of L-tyrosine by the replacement of nascent genetic elements of biosynthesis enzymes with synthetic promoters and a 5′-UTR for maximum expression. Using UTR Designer, the carbon flux at the PEP node was further optimized for maximum productivity by testing the effect of various expression levels of PEP synthetase gene *(ppsA)* on L-tyrosine yield. Using this approach, an *E. coli* strain that produces recombinant L-tyrosine at 3 g/L, which is higher than that produced by the wild-type strain, was generated. This work demonstrates that pathway optimization by UTR redesign can be an effective strategy for increasing the yield in recombinant L-tyrosine production.

## Results and Discussion

### Rational engineering of L-tyrosine overproduction pathway

In *E. coli*, the elements of the L-tyrosine synthetic pathway are encoded by eleven genes. The first step of this pathway consists of the condensation of erythrose-4-phosphate (E4P) and PEP to yield 3-deoxy-D-arabino-heptulosonate-7-phosphate (DAHP) by DAHP synthase (AroG). DAHP is then converted to chorismate (CHA) via six enzymatic reactions catalyzed by 3-dehydroquinate synthase (AroB), 3-dehydroquinate dehydratase (AroD), shikimate dehydrogenase (AroE), shikimate kinase II (AroL), 5-enolpyruvylshikimate-3-phosphate synthase (AroA), and CHA synthase (AroC). Tyrosine is then finally synthesized from CHA by chorismate mutase, prephenate dehydrogenase (TyrA), and transaminase (TyrB). In the tyrosine synthetic pathway, AroG, AroB, and TyrA are negatively regulated by L-tyrosine^41^,^42^. In addition, the TyrR protein, a regulator expressed in response to intracellular tyrosine concentrations, tightly regulates the expression of genes such as AroL, TyrA, and TyrB^13^,^43^.

**Figure 1:**
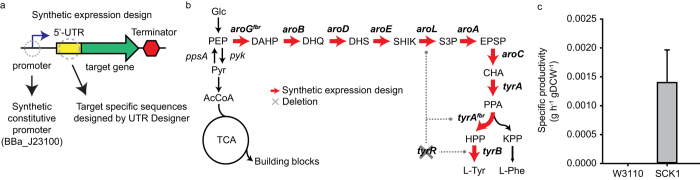
The L-tyrosine biosynthetic pathway engineering strategy. (**a**) Each gene was under the control of synthetic expression design that substitutes native promoter and 5′-UTR with synthetic constitutive promoter and designed 5′-UTR specific to target gene. (**b**) Dashed lines indicate feedback regulation, ‘X’ denotes deletion of the *tyrR* gene, and thick red arrows represent overexpression of genes in the L-tyrosine synthetic pathway. Abbreviations: Glc, glucose; PEP, phosphoenolpyruvate; Pyr, pyruvate; AcCoA, acetyl-CoA; DAHP, 3-deoxy-D-arabino-heptulosonate-7-phosphate; DHQ, 3-dehydroquinate; DHS, 3-dehydroshikimate; SHIK, shikimate; S3P, shikimate-3-phosphate; EPSP, 5-enolpyruvylshikimate-3-phosphate; CHA, chorismate; PPA, prephenate; HPP, 3-hydroxyphenylpyruvate; L-tyr, L-tyrosine; KPP, keto-phenylpyruvate; L-Phe, L-phenylalanine. (**c**) Comparison of L-tyrosine productivity of wild-type (W3110) and rationally engineered (SCK1) *E. coli* strain. The specific productivity of L-tyrosine was increased to 0.0014 g/h/g DCW in the SCK1 strain while the wild type strain did not produce L-tyrosine. The y-axis represents specific productivity of L-tyrosine (g/h/g DCW) in each strain. Each point and error bar indicates means and standard deviations between measurements from biological triplicate cultures.

To construct an L-tyrosine-overproducing strain (SCK1), a strong and constitutive promoter, BBA_J23100 from the Registry of Standard Biological Parts (http://partsregistry.org), was switched with each original promoter to increase the transcription levels of all genes in the L-tyrosine biosynthetic pathway. Additionally, a 5′-UTR sequence was designed by UTR Designer to achieve the maximum expression level of each gene in the tyrosine pathway (Fig. 1a). Based on the N-terminal coding sequence of each gene, UTR Designer generated different 5′-UTR sequences that can obtain maximum expression levels (Supplementary Table S1). Since the 5′-UTR sequences can be different depending on coding sequences that we input, we selected the sequence with the highest expression level for each gene and replaced it from the original UTR sequence of the gene on a chromosome using Red recombination. In case of *aroB* where we could not achieve high translation rate with native coding sequence, we further optimized the coding sequence with same codon preference to achieve high translation rate (Supplementary Table S1). Thus, each gene in the pathway was expressed as a monocistronic transcript under the control of the synthetic constitutive promoter and the 5′-UTR using inherent terminator (Fig. 1b). The *tyrR* gene encoding the TyrR protein was additionally knocked out to remove transcriptional regulation of the L-tyrosine pathway. Feedback-resistant variants of AroG^fbr^ [D146N] and TyrA^fbr^ [M53I; A354V] were then substituted for the wild-type enzymes to deregulate feedback inhibition by L-tyrosine^8^. In other studies, overexpression was performed using inducible promoter such as T7 promoter on a plasmid and only several target genes were engineered among all genes involved in tyrosine biosynthetic pathway^8^,^44^,^45^,^46^. However, in this study, each gene was under the control of constitutive strong promoters and synthetic 5′-UTRs on the chromosome to eliminate problems related with marker and plasmid origin incompatibilities as well as to eliminate the need for antibiotics and inducers^15^.

The rationally engineered *E. coli* strain SCK1 was cultivated in M9 minimal medium to allow for the L-tyrosine productivity of the strain to be determined. The SCK1 strain exhibited an L-tyrosine productivity of 0.0014 g/h/g dry cell weight (DCW), while the wild-type W3110 *E. coli* strain produced negligible levels of L-tyrosine (Fig. 1c). This indicates that the rationally maximized expression levels of specific genes at both the transcription and translation levels increases L-tyrosine production as a result of linear flux amplification in the L-tyrosine biosynthetic pathway.

### Optimization of L-tyrosine biosynthetic pathway by fine-tuning expression level of *ppsA*

**Figure 2:**
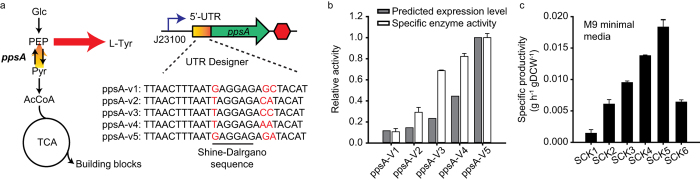
Carbon flux redistribution at the PEP node by fine-tuning of *ppsA* expression. (**a**) Pathway optimization for fine-tuning the expression levels of the *ppsA* gene using UTR Designer. J23100 indicates a strong constitutive promoter from the Registry of Standard Biological Parts (BBa_J23100; http://partsregistry.org). (**b**) Comparisons of predicted expression levels from UTR Designer and specific enzyme activities of *ppsA* variants. (**c**) The specific L-tyrosine productivity of each *ppsA* variant after 24 h cultivation in M9 minimal medium. Each point and error bar indicates means and standard deviations between measurements from biological triplicate cultures.

Although L-tyrosine productivity of *E. coli* was enhanced by amplification of the L-tyrosine biosynthetic pathway, we hypothesized that maximum L-tyrosine production would not be achieved by this method because of the metabolic imbalance between cell growth and L-tyrosine production around the PEP node. PEP is a starting material in the L-tyrosine pathway and is also used as precursor of pyruvate that enters into the TCA cycle (Fig. 2a). Accordingly, an excessive increase of flux toward the L-tyrosine pathway results in a decreased L-tyrosine productivity by cells due to growth inhibition caused by reduced flux to the TCA cycle. Optimization of flux distribution at the PEP node must therefore be optimized in order for maximum L-tyrosine productivity to be achieved.

To optimize flux distribution at the PEP node, the *ppsA* gene (encoding PEP synthetase) was selected as the regulation target because it has been shown that the overexpression of *ppsA* increases tyrosine production by *E. coli*^8^,^11^,^47^. The growth of a *ppsA*-overexpressing strain could, however, be inhibited compared to that of a wild type strain because of decreased metabolic flux to the TCA cycle at PEP node, which causes decrease of L-tyrosine productivity despite of increase of flux toward L-tyrosine synthesis. Indeed, this was found to be the case when the growth rate of a *ppsA*-overexpressing strain was measured in this study (Supplementary Table S2). It means that overexpression or knock out of PpsA is not enough to optimize flux distribution at PEP node for maximum L-tyrosine production. For this reason, optimization of carbon flux at the PEP node through precise control of the expression level of *ppsA* in both transcription and translation levels is essential for obtaining maximum L-tyrosine production. To precisely regulate the expression levels of genes, it is often not enough to control expression at a transcriptional level alone, as this does not control expression at a translational level especially when additional regulatory elements such as riboswitches are present. Furthermore, optimal catalytic activity, in many cases, cannot be easily achieved by modulating the promoter strength only. Accordingly, for maximum tyrosine production to be achieved, the expression level needs to be controlled at a translational level as well as at a transcriptional level.

Toward the optimization of flux distribution around the PEP node, we input maximum expression level at UTR Designer for *ppsA* and obtained a 5′-UTR sequence for this expression level (TTAACTTTAAT*G*AGGAG*AA*ATACAT). From this particular sequence, we selected three nucleotides around the Shine-Dalgarno (SD) sequence that significantly caused expression changes upon single base pair mutation in the model. We intended to design degenerate 5′-UTR sequences (TTAACTTTAAT*K*AGGAG*NM*ATAC -AT) that yield 16 different sequences. Unfortunately, we could only obtain five variants (pPpsA-V1 – pPpsA-V5) from our cloning steps (Fig. 2a). Their predicted expression levels were different due to the changes in both 16S rRNA binding affinity and mRNA structures (Supplementary Table S3). Expression cassettes yielding different *ppsA* expression levels from synthetic promoter and designed 5′-UTR sequences were replaced with original promoter and 5′ UTR of *ppsA* gene on the chromosome in rationally engineered SCK1 *E. coli* strain. To verify the actual *ppsA* expression levels, the specific activity of *ppsA* was measured for each variant. Notably, the specific activity of each variant highly correlated with the predicted *ppsA* expression level (Fig. 2b and Supplementary Fig. S1). The L-tyrosine productivity of each of these five variants was subsequently measured to assess the effect of flux redistribution at the PEP node on L-tyrosine yield. As shown in Fig. 2c, the L-tyrosine productivities of the *ppsA* variants were higher than that of the original engineered strain (SCK1), but the trend of L-tyrosine productivity differed from that of *ppsA* enzyme activity. Although specific *ppsA* activity was higher in the SCK6 strain than in the SCK5 strain, L-tyrosine productivity was dramatically lower in the SCK6 strain than in the SCK5 strain (Fig. 2c). This result indicates that an optimal *ppsA* expression level for maximum tyrosine productivity does exist, and that optimal flux distribution is achieved by fine control of gene expression at both transcription and translation levels. These findings furthermore demonstrate that blindly maximizing the expression level of *ppsA* is not an effective way of reaching maximum L-tyrosine productivity as this blind approach causes a metabolic imbalance at a branch node.

### Optimization of culture condition for L-tyrosine overproduction

**Figure 3:**
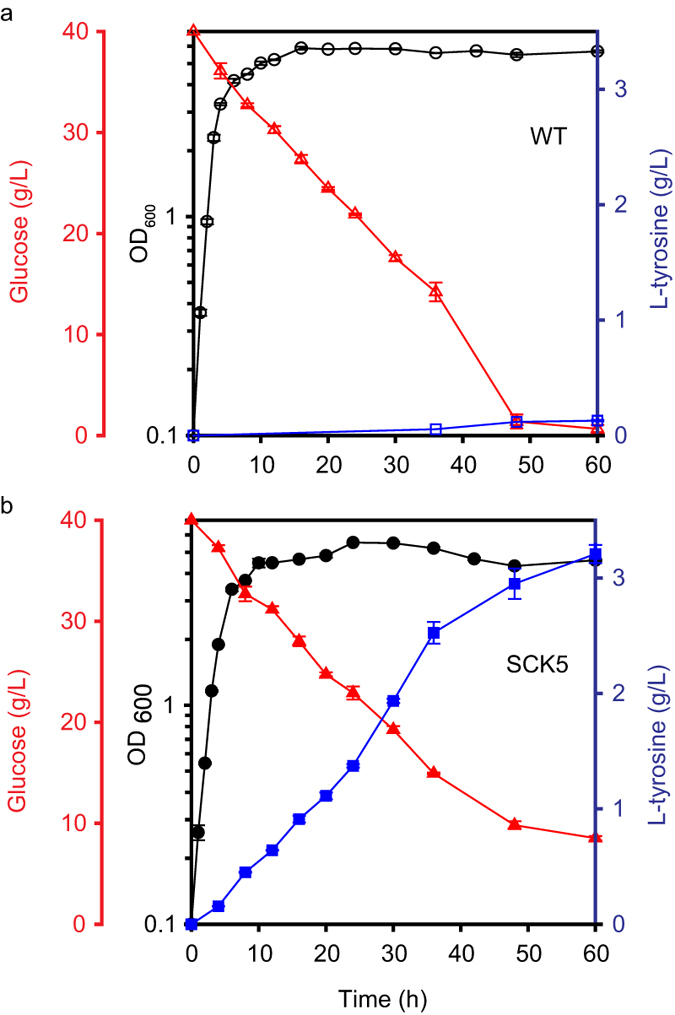
Fermentation profiles of *E. coli* strains cultivated in complex medium. Data for the (**a**) wild-type (W3110; open symbols) and (**b**) SCK5 (closed symbols) *E. coli* strains are shown. pH adjustments were made at 6-h intervals. The left y-offset and right y-axis represent glucose and L-tyrosine concentrations (g/L), respectively. The left y-axis represents OD_600_ and the x-axis represents culture time (h). Each point and error bar indicates means and standard deviations between measurements from biological triplicate cultures. Symbols: circle, OD_600_; triangle, glucose; rectangle, L-tyrosine.

Although metabolic flux was optimized by controlling the expression level of *ppsA*, maximal L-tyrosine production in *E. coli* also requires optimization of the culture conditions. Cell growth and carbon uptake are inhibited in media with a low pH (resulting from the accumulation of L-tyrosine). For this reasons, the high L-tyrosine-producing *E. coli* strain engineered in this study (SCK5) produced only up to 0.3265 g/L L-tyrosine in batch cultures grown in M9 minimal medium. With pH control, L-tyrosine production was increased to 0.5155 g/L (Supplementary Table S2). We further tested the SCK5 *E. coli* strain as well as the other engineered *ppsA* variants in complex medium, after which L-tyrosine production was measured. As in the case of M9 minimal medium, L-tyrosine productivity among the *ppsA* variants grown in complex medium was highest in the SCK5 strain (Supplementary Fig. S2) and the highest titer was 1 g/L. After culturing in complex medium with pH adjustment, the L-tyrosine titer and productivity increased to 3 g/L, 0.0354 g/h/g DCW and 0.102 g L-tyrosine/g glucose yield from the SCK5 strain (Fig. 3b) compared with negligible L-tyrosine production by the wild type W3110 *E. coli* strain (Fig. 3a). However, glucose uptake by the SCK5 strain was reduced after ~48 h in culture when L-tyrosine started to be precipitated. We are not sure why glucose uptake is inhibited after ~48 h. One possibility is that tyrosine precipitation may be accompanied by cell aggregation^48^ and reduce the performance of the batch culture. Collectively, our findings indicate that L-tyrosine was produced to capacity in the engineered SCK5 *E. coli* strain, and that this capacity is limited by low pH as a result of L-tyrosine accumulation.

In this study, we demonstrate that flux redistribution around the PEP node, the junction between flux to the TCA cycle and flux to the L-tyrosine synthetic pathway, is one of the most important factors to consider in engineered strain improvement for maximum L-tyrosine production. Pathway optimization using UTR Designer to achieve precise quantification of expression was also shown to contribute greatly to optimal L-tyrosine productivity of *E. coli*. This engineered *E. coli* strain for L-tyrosine production could be further applied to produce flavonoids by introducing several pathway enzymes in the flavonoid synthetic pathway^49^.

One of the major challenges associated with metabolic engineering is achieving precise control and optimized expression levels of genes to yield large amounts of metabolites of interest. Our findings show that UTR Designer is a useful tool by which fine control of protein expression can be gained for pathway optimization. Our previous work on the optimization of redox balance, genetic circuit construction, and control of heterologous expression^32^,^36^ also demonstrates that fine control of carbon flux redistribution can be achieved using UTR Designer.

Recently, we also developed UTR Library Designer with same biophysical model to generate UTR library covering large combinatorial space of expression level and demonstrated its usefulness for pathway engineering through lysine and hydrogen production^37^. We believe that both UTR Designer and UTR Library Designer can thus be used as efficient tools by which pathways can be optimized for the production of various chemicals, fuels, and recombinant proteins.

## Methods

### Bacterial strains, plasmids, and primers

The *E. coli* bacterial strains, plasmids, and oligonucleotides used in this study are listed in Supplementary Table S4 and S5. Phusion polymerase and restriction endonucleases were purchased from New England Biolabs (Beverly, MA, USA) and the oligonucleotides used were synthesized by Genotech (Daejeon, South Korea) and Bioneer (Daejeon, South Korea). Other reagents were obtained from Sigma-Aldrich (St. Louis, MO).

To construct the pTyrA^*fbr*^ vector, the *tyrA* gene was amplified by polymerase chain reaction (PCR) using V-tyrA-F/R primers and was then inserted into a pMD20-T vector. The *tyrA*^*fbr*^ gene was constructed by site-directed mutagenesis using the M-tyrA-F1/R1 and -F2/R2 primer pairs. A tyrA^*fbr*^-*FRT-Kan*^*R*^*-FRT* fragment was amplified by overlap PCR by using the P-tyrA-F/R and P-FKF-F/R primers and the pGFKF2 and pTyrA^*fbr*^ vectors, after which it was inserted into a pMD20-T vector. To construct the pAroG^*fbr*^ vector, the *aroG* fragment amplified using the V-aroG-F/R primer set was inserted into a pMD20-T vector, after which feedback inhibition of AroG was stopped by site-directed mutagenesis using the M-aroG-F/R primer pair. The aroG^*fbr*^ vector was subsequently inserted into the pGFKF2 vector using the *Sac*I and *Kpn*I restriction sites.

All chromosomal manipulations were carried out using the Red recombination system with the pKD46 and pCP20 plasmids as described in previous studies^50^,^51^. To construct the SCK1 *E. coli* strain, *tyrR* was deleted by insertion of the *FRT-Kan*^*R*^*-FRT* fragment amplified with the D-tyrR-F/D-tyrR-R primer pair and by replacement of the native promoter and 5′-UTR of *tktA, aroA*, *aroB*, *aroC, aroD, aroE, aroL*, and *tyrB* with a BBA_J23100 promoter and a redesigned 5′-UTR, respectively, by using an *FRT-Kan*^*R*^*-FRT* fragment amplified by each corresponding forward/reverse primer pair. The native *aroG* and *tyrA* genes were then replaced by *aroG*^*fbr*^ and *tyrA*^*fbr*^ with a BBA_J23100 promoter and a redesigned 5′-UTR by using an *FRT-Kan*^*R*^*-FRT* fragment amplified by the O-aroG-F/O-aroG-R and O-tyrA-F/O-tyrA-R primer pairs, respectively. To verify the integrity of the genomic modifications, we performed sequencing after recombination.

To modulate *ppsA* expression, five different 5′-UTR sequences were designed using UTR Designer (http://sbi.postech.ac.kr/utr_designer)^32^. To construct the SCK2– SCK6 strains, the native promoter and 5′-UTR of *ppsA* was replaced with a BBA_J23100 promoter and a rationally redesigned 5′-UTR sequence (*ppsA* (v1–5)) using an *FRT-Kan*^*R*^*-FRT* fragment amplified by the O-ppsA-F/O-ppsA-R primer pair.

### Cell cultures and growth measurement

The wild type and engineered *E. coli* strains were cultivated in either M9 minimal medium (M9 salt solutions; Sigma-Aldrich, St. Louis, MO; 5 mM MgSO_4_, 0.1 mM CaCl_2_, 5 or 40 g/L glucose, and appropriate antibiotics) or complex medium (6.75 g KH_2_PO_4_, 2 g (NH_4_)_2_HPO_4_, 0.85 g citric acid, 3 g yeast extract, 40 g glucose, 10 mL trace metal solution per liter [10 g FeSO_4_.7H_2_O, 2.2 g ZnSO_4_.7H_2_O, 0.58 g MnSO_4_.4H_2_O, 1 g CuSO_4_.5H_2_O, 0.1 g (NH_4_)_6_Mo_7_O_24_.4H_2_O, 0.2 g Na_2_B_4_O_7_.10H_2_O, and 10 mL 35% HCl per liter], and appropriate antibiotics; pH 6.8).

Cells were cultured at 37 °C with shaking (200 rpm) and cell densities of *E. coli* cultures were measured at a wavelength of 600 nm (OD_600_) using a UV-1700 spectrophotometer (Shimadzu, Kyoto, Japan).

Seed cultures were prepared by inoculating colonies from an LB plate into 3 mL M9 minimal medium or complex medium. After adaptation, this initial seed culture was used to inoculate 3 mL of the same medium. When these second-round seed cultures reached an OD_600_ of 0.8–1.0, they were washed twice and individually inoculated either into 25 mL M9 minimal medium in a 300 mL flask at an OD_600_ of 0.05 or into 50 mL complex medium in a 300 mL flask at an OD_600_ of 0.1. All cell culture experiments were conducted in biological triplicates. The pH of culture medium was adjusted to 6.8 at 6-h intervals using 10 M NaOH.

### PEP synthetase (PpsA) activity assay

To test PpsA activity, cells with different expression levels of *ppsA* were harvested in mid-log phase and then washed with cold phosphate-buffered saline (PBS). The resulting cell pellets were resuspended and lysed with Bug Buster Master Mix (EMD Bioscience, San Diego, CA, USA) supplemented with protease inhibitor cocktail (Sigma, St. Louis, MO, USA) according to the manufacturer’s instructions. Total protein concentration in cell lysates was determined using the Bradford assay-based Bio-Rad Protein Assay Dye (Bio-Rad, Hercules, CA, USA) with bovine serum albumin as a standard. PpsA activity was measured according to a previously described method^52^. The assay mixture contained 4 μmol/L pyruvate, 10 μmol/L ATP, 10 μmol/L MgCl_2_, and 100 μmol/L Tris-HCl (pH 8.0). Assay reactions were initiated by the addition of 40 μL crude lysate into the assay mixture, after which reaction mixtures were incubated at 30 °C for 5 in. Reactions were terminated by the addition of aliquots (20 μL) of the reaction mixture into a mixture containing 0.066 mL 0.1% aqueous solution of 2,4-dinitrophenylhydrazine and 0.18 mL H_2_O followed by incubation at 30 °C for 10 min. The resulting mixture was further incubated with 0.334 mL 10% (w/v) NaOH at 30 °C for 10 min. PpsA activity was determined by measuring the depletion of pyruvate at 445 nm using a VICTOR^3^ 1420 Multilabel Counter (PerkinElmer, Waltham, MA, USA). PpsA activity was normalized to total protein content in cell lysates to obtain specific PpsA activity (U/mg).

### L-Tyrosine production and detection of metabolites

The concentration of glucose consumed by the *E. coli* strains was determined by high-performance liquid chromatography (HPLC) using an Aminex HPX-87H column (Bio-Rad Laboratories, Richmond, CA, USA) at a flow rate of 0.6 mL/min at 65 °C using 5 mM H_2_SO_4_ as the mobile phase. The glucose concentration was monitored using a Shodex RI-101 detector (Shodex, Klokkerfaldet, Denmark). L-tyrosine concentrations in culture broth were determined using a pre-column o-phthalaldehyde derivatization method coupled with reversed-phase liquid column chromatography (Acclaim 120 C18; Dionex, Sunnyvale, CA, USA) using an UltiMate 3000 analytical HPLC system (Dionex). Derivatized L-tyrosine was eluted at a flow rate of 1.5 mL/min with a gradient of an acetonitrile, methanol, and water solution (45:45:10; % [v/v]) and 50 mM sodium acetate buffer (pH 6.5). An ultraviolet-visible (UV-Vis) diode array detector was used to detect derivatized L-tyrosine at 338 nm.

## Additional Information

**How to cite this article**: Kim, S. C. *et al.* Pathway optimization by re-design of untranslated regions for L-tyrosine production in *Escherichia coli*. *Sci. Rep.* **5**, 13853; doi: 10.1038/srep13853 (2015).

## Supplementary Material

Supplementary Information
