# Supplementary material for: Pathway optimization by re-design of untranslated regions for L-tyrosine production in Escherichia coli
Source: Sci Rep. 2015 Sep 8;5:13853. doi: 10.1038/srep13853 (PMC4561953; doi:10.1038/srep13853)
Supplement: Supplementary Information [file srep13853-s1.doc]

**Supplementary Data for:**

**Pathway optimization by re-design of untranslated regions for L-tyrosine production in *Escherichia coli***

Seong Cheol Kim^1^, Byung Eun Min^1^, Hyun Gyu Hwang, Sang Woo Seo^2^, Gyoo Yeol Jung^2^

^1^ These authors contributed equally to this work.

2 To whom correspondence should be addressed.

E-mail: gyjung@postech.ac.kr (G. Y. J.); [swseo84@gmail.com](mailto:swseo84@gmail.com) (S. W. S)

**Contents:**

**Supplementary Tables S1-S5**

**Supplementary Figures S1-S2**

**Supplementary Tables**

**Supplementary Table S1. Redesign of 5’-UTRs for genes in tyrosine synthesis pathway.**

| **Gene** | **Synthetic 5’-UTR** | **N-terminal 35 bp** | **dG_SD_** | **dG_start_** | **dG_spacing_** | **dG_direct_** | **dG_indirect_** | **dG_UTR_** | **Predicted Expression Level** |
| --- | --- | --- | --- | --- | --- | --- | --- | --- | --- |
| *aroG* | GACTATTTCAAAAGGAGCATCACGA | ATGAATTATCAGAACGACGATTTACGCATCAAAGA | -10.38 | -1.19 | 0.29 | -2.1 | -4.4 | -8.04 | 713468.88 |
| *aroB* | GAAGCAGAGTTAAGGAGGAGAACAT | ATGGAGCGTATTGTCGTTACTCTGGGCGAACGTAG | -14.88 | -1.19 | 0.29 | -1.5 | -5.7 | -12.19 | 7267981.04 |
| *aroD* | AGGCCCGAAATAAGGAGGATCATTC | ATGAAAACCGTAACTGTAAAAGATCTCGTCATTGG | -14.88 | -1.19 | 0.29 | -2.5 | -1.3 | -13.89 | 18808117.19 |
| *aroE* | GCCGATCAAATAAGGAGGAACTCGA | ATGGAAACCTATGCTGTTTTTGGTAATCCGATAGC | -14.88 | -1.19 | 0.29 | -1.9 | -5.7 | -11.99 | 6498806.48 |
| *aroL* | GAACCAGCAATAAGGAGGAACTCGC | ATGACACAACCTCTTTTTCTGATCGGGCCTCGGGG | -14.88 | -1.19 | 0.29 | -5.3 | -3.6 | -11.34 | 4518018.63 |
| *aroA* | CACCCCTAGATAAGGAGCATCTCAT | ATGGAATCCCTGACGTTACAACCCATCGCTCGTGT | -10.98 | -1.19 | 0.29 | -2.4 | -1.3 | -10.04 | 2183618.89 |
| *aroC* | CACGATAGGAGAAGGAGGTAGATAT | ATGGCTGGAAACACAATTGGACAACTCTTTCGCGT | -15.08 | -1.19 | 0.29 | -2 | -2 | -13.99 | 19890028.82 |
| *tktA* | CACGCTAAATTAAGGAGCCCCAAAC | ATGTCCTCACGTAAAGAGCTTGCCAATGCTATTCG | -10.88 | -1.19 | 0.29 | -1.5 | -2.6 | -9.74 | 1846318.29 |
| *tyrA* | CCGAAGACGCTAAGGAGGTACGAGA | ATGGTTGCTGAATTGACCGCATTACGCGATCAAAT | -15.08 | -1.19 | 0.29 | -1.9 | -6.6 | -11.74 | 5650779.74 |
| *tyrB* | TTCTGAACCTCCTTTGCTATAGTAC | GTGTTTCAAAAAGTTGACGCCTACGCTGGCGACCC | -14.68 | -0.08 | 0.29 | -0.6 | -7.8 | -10.27 | 2484774.33 |

The coding sequence of *aroB* has also been redesigned with same codon preference.

**Supplementary Table S2.** Fermentation data of *ppsA* variants in M9 minimal medium

| **Strain** | **pH control** | **Specific growth rate**  **(/h)** | **L-tyrosine titer**  **(g/L)** | **Yield**  **(g L-tyrosine/g glucose)** | **Specific productivity**  **(g /h/g DCW)** |
| --- | --- | --- | --- | --- | --- |
| SCK2 | No | 0.5267 ± 0.0037 | 0.1034 ± 0.0037 | 0.0207 ± 0.0007 | 0.0061 ± 0.0005 |
| SCK3 | No | 0.5242 ± 0.0033 | 0.1950 ± 0.0051 | 0.0390 ± 0.0010 | 0.0095 ± 0.0002 |
| SCK4 | No | 0**.**4102 ± 0.0041 | 0.2824 ± 0.0022 | 0.0565 ± 0.0004 | 0.0138 ± 0.0001 |
| SCK5 | No | 0.3762 ± 0.0018 | 0.3265 ± 0.0129 | 0.0653 ± 0.0026 | 0.0183 ± 0.0008 |
| SCK6 | No | 0.4480 ± 0.0000 | 0.1487 ± 0.0112 | 0.0297 ± 0.0022 | 0.0064 ± 0.0064 |
| SCK5 | Yes | 0.3941 ± 0.0027 | 0.5155 ± 0.1689 | 0.0743 ± 0.0013 | 0.0194 ± 0.0005 |

**Supplementary Table S3. Redesign of 5’-UTRs for *ppsA*.**

| **Cloned** | **Synthetic 5’-UTR** | **N-terminal 35 bps** | **dG_SD_** | **dG_start_** | **dG_spacing_** | **dG_direct_** | **dG_indirect_** | **dG_UTR_** | **Predicted Expression Level** |
| --- | --- | --- | --- | --- | --- | --- | --- | --- | --- |
|  | TTAACTTTAATTAGGAGAGCTACAT | ATGTCCAACAATGGCTCGTCACCGCTGGTGCTTTG | -9.48 | -1.19 | 0.29 | -6.6 | -9 | -2.59 | 33850.5 |
| *ppsA-v1* | TTAACTTTAATGAGGAGAGCTACAT |  | -11.78 | -1.19 | 0.29 | -7 | -9 | -4.69 | 109561.26 |
| *ppsA-v2* | TTAACTTTAATTAGGAGACATACAT |  | -9.48 | -1.19 | 0.29 | -3.9 | -6.7 | -5.09 | 137030.54 |
|  | TTAACTTTAATTAGGAGATATACAT |  | -9.48 | -1.19 | 0.29 | -5.1 | -4.8 | -5.44 | 166660.5 |
| *ppsA-v3* | TTAACTTTAATTAGGAGACCTACAT |  | -9.48 | -1.19 | 0.29 | -4 | -4.9 | -5.94 | 220436.31 |
|  | TTAACTTTAATTAGGAGAGATACAT |  | -9.48 | -1.19 | 0.29 | -3.5 | -4.8 | -6.24 | 260707.43 |
|  | TTAACTTTAATTAGGAGATCTACAT |  | -9.48 | -1.19 | 0.29 | -3.7 | -3.9 | -6.59 | 317079.91 |
|  | TTAACTTTAATGAGGAGACATACAT |  | -11.78 | -1.19 | 0.29 | -4.9 | -6.7 | -6.89 | 375006.67 |
| *ppsA-v4* | TTAACTTTAATTAGGAGAAATACAT |  | -9.48 | -1.19 | 0.29 | -2.7 | -3.9 | -7.09 | 419391.07 |
|  | TTAACTTTAATTAGGAGAACTACAT |  | -9.48 | -1.19 | 0.29 | -2.7 | -3.9 | -7.09 | 419391.07 |
|  | TTAACTTTAATGAGGAGATATACAT |  | -11.78 | -1.19 | 0.29 | -5.1 | -4.8 | -7.74 | 603260.33 |
|  | TTAACTTTAATGAGGAGACCTACAT |  | -11.78 | -1.19 | 0.29 | -4 | -4.9 | -8.24 | 797912.4 |
| *ppsA-v5* | TTAACTTTAATGAGGAGAGATACAT |  | -11.78 | -1.19 | 0.29 | -3.5 | -4.8 | -8.54 | 943681.6 |
|  | TTAACTTTAATGAGGAGAAATACAT |  | -11.78 | -1.19 | 0.29 | -3.7 | -3.9 | -8.89 | 1147732.83 |
|  | TTAACTTTAATGAGGAGAACTACAT |  | -11.78 | -1.19 | 0.29 | -2.7 | -3.9 | -9.39 | 1518068.1 |
|  | TTAACTTTAATGAGGAGAAATACAT |  | -11.78 | -1.19 | 0.29 | -2.7 | -3.9 | -9.39 | 1518068.1 |

We only successfully cloned 5 variants out of 16 and used them for further experiments.

**Supplementary Table S4. Strains and plasmids used in this study.**

| **Name** | **Relevant characteristics** | **Source** |
| --- | --- | --- |
| **Strains** |  |  |
| Mach1-T1^R^ | F^-^ φ80(*lac*Z)ΔM15 Δ*lac*X74 *hsd*R(r_K_-mK+) Δ*rec*A1398 *end*A1 *ton*A | Invitrogen |
| W3110 | F^-^ λ^-^ rph-1 IN(*rrnD*, *rrnE*)1 | ATCC 27325 |
| SCK1 | W3110 Δ*tyrR* *aroG*:: PBBa_J23100-synUTR_aroG_-*aroG^fbr^* *tyrA*:: PBBa_J23100-synUTR_tyrA_-*tyrA^fbr^* P_aroABCDELtyrB_-UTR_aroABCDELtyrB_:: PBBa_J23100-synUTR_aroABCDELtyrB_ | This study |
| SCK2 | SCK1 P_ppsA_-UTR_ppsA_:: PBBa_J23100-synUTR_ppsA(V1)_ | This study |
| SCK3 | SCK 1 P_ppsA_-UTR_ppsA_:: PBBa_J23100-synUTR_ppsA(V2)_ | This study |
| SCK4 | SCK 1 P_ppsA_-UTR_ppsA_:: PBBa_J23100-synUTR_ppsA(V3)_ | This study |
| SCK5 | SCK 1 P_ppsA_-UTR_ppsA_:: PBBa_J23100-synUTR_ppsA(V4)_ | This study |
| SCK6 | SCK 1 P_ppsA_-UTR_ppsA_:: PBBa_J23100-synUTR_ppsA(V5)_ | This study |
|  |  |  |
| **Plasmids** |  |  |
| pKD46 | Red recombinase expression vector; AmpR | (Datsenko &  Wanner, 2000) |
| pCP20 | FLP expression vector; AmpR | (Datsenko &  Wanner, 2000) |
| pGEM | Cloning vector, Amp^R^, | Promega |
| pMD20-T | Cloning vector, Amp^R^ | Takara |
| pGFKF2 | pGEM-FRT-*Kan^R^*-FRT-KpnI-SacI | (Yang J, 2013) |
| pTyrA | pMD20-P_BBA J23100_-synUTR_tyrA_-*tyrA* | This study |
| pTyrA^fbr^ | pMD20-P_BBA J23100_-synUTR_tyrA_-*tyrA^fbr^*-FRT- *Kan^R^*-FRT | This study |
| pAroG^fbr^ | pGFKF2-*KpnI*-P_BBA_ _J23100_-synUTR_aroG_-*aroG^fbr^*-*SacI* | This study |
| pPpsA-V1 | pMD20-FRT-*Kan^R^*-FRT-PBBA_J23100-synUTR_ppsA(V1)_ | This study |
| pPpsA-V2 | pMD20-FRT-*Kan^R^*-FRT-PBBA_J23100-synUTR_ppsA(V2)_ | This study |
| pPpsA-V3 | pMD20-FRT-*Kan^R^*-FRT-PBBA_J23100-synUTR_ppsA(V3)_ | This study |
| pPpsA-V4 | pMD20-FRT-*Kan^R^*-FRT-PBBA_J23100-synUTR_ppsA(V4)_ | This study |
| pPpsA-V5 | pMD20-FRT-*Kan^R^*-FRT-PBBA_J23100-synUTR_ppsA(V5)_ | This study |
|  |  |  |

**Supplementary Table S5.** Primers used in this study.

| **Name** | **Sequence (5**′**-3**′**)^a,b,c^** |
| --- | --- |
| **O-aroL-F** | gcggagctggagaagtggtggctggaagtgcaacgtagtcgtggctaaatgatcgtgcgaacgacctgct |
| **O-aroL-R1** | gcgagttcctccttattgctggttcgctagcactgtacctaggactgagctagccgtcaacagtagtcgagtcgctccga |
| **O-aroL-R2** | gttgttttaccacagccccgaggcccgatcagaaaaagaggttgtgtcatgcgagttcctccttattgctggt |
| **C-aroL-F** | cctttggctgctgaagccatcg |
| **C-aroL-R** | gtggatggcgcagttaccgc |
| **O-aroA-F** | gttcgaacgccgtcacggttaatgccgaaattttgcttaatccccacagcgatcgtgcgaacgacctgct |
| **O-aroA-R1** | atgagatgctccttatctaggggtggctagcactgtacctaggactgagctagccgtcaacagtagtcgagtcgctccga |
| **O-aroA-R2** | ttaatagtgccatcgacacgagcgatgggttgtaacgtcagggattccatatgagatgctccttatctaggggtg |
| **C-aroA-F** | ctggccttcatgcactgaaaggtc |
| **C-aroA-R** | cgtcatcgctatccagcagattgg |
| **O-aroA-F1** | gttcgaacgccgtcacggttaatgccgaaattttgcttaatccccacagcgcatgaccggcgcgatgc |
| **O-aroA-R3** | atgagatgctccttatctaggggtggctagcactgtacctaggactgagctagccgtcaagctcagcggatctcatgcgc |
| **O-aroC-F** | gcgatttataaagattaagtaaacacgcaaacacaacaataacggagccg-ccgcatgaccgcgcgatgc |
| **O-aroC-R1** | ctacctccttctcctatcgtgggctagcactgtacctaggactgagctagccgtcaacgcgacgacaggcacatgcg |
| **O-aroC-R2** | tcgccgaaggtggttacgcgaaagagttgtccaattgtgtttccagccatatatctacctccttctcctatcgtgggct |
| **C-aroC-F** | ggcgatggtgtgtttatgctcacc |
| **C-aroC-R** | cggagagaattttgacctgatccgg |
| **O-tyrB-F** | ctctggcggatgtacgtttgtcatgagtctcactctgttgctaattgccgccgcatgaccgcgcgatgc |
| **O-tyrB-R1** | ctgaacctcctttgctatagtacgctagcactgtacctaggactgagctagccgtcaacgcgacgacaggcacatgcg |
| **O-tyrB-R2** | ataagcgtaagaatcgggtcgccagcgtaggcgtcaactttttgaaacacttctgaacctcctttgctatagtacgc |
| **C-tyrB-F** | cgcgcctgacttcaagggtc |
| **C-tyrB-R** | gccatgaggctgcgcattcag |
| **O-aroE-F** | cttcagaaatccgcgatgccctgacgggtgaactgtttcgacaggggtaagatcgtgcgaacgacctgct |
| **O-aroE-R1** | gttcctccttatttgatcggcgctagcactgtacctaggactgagctagccgtcaacagtagtcgagtcgctccga |
| **O-aroE-R2** | ggcgatttgctgtgggctatcggattaccaaaaacagcataggtttccattcgagttcctccttatttgatcggcgc |
| **C-aroE-F** | ggtggttgctttgtgccaggc |
| **C-aroE-R** | gctcatccgctctggcaaaagc |
| **O-aroD-F** | gatttccctctggaatatgttaaacaggtcatggggttcggtgcctgacaccgcatgaccgcgcgatgc |
| **O-aroD-R1** | gaatgatcctccttatttcgggcctgctagcactgtacctaggactgagctagccgtcaacgcgacgacaggcacatgcg |
| **O-aroD-R2** | ttaggtgcgcccgtaccaatgacgagatcttttacagttacggttttcatgaatgatcctccttatttcgggcctg |
| **C-aroD-F** | gacgaagttattgcagcaggcgc |
| **C-aroD-R** | cgccatgacagactccacattgg |
| **O-aroB-F** | ggctttatatacactcgtctgcgggtacagtaattaaggtggatgtcgcggcatgaccggcgcgatgc |
| **O-aroB-R1** | ccttaactctgcttc-gctagcactgtacctaggactgagctagccgtcaa-gctcagcggatctcatgcgc |
| **O-aroB-R2** | gggtagctacgttcgcccagagtaacgacaatacgctccatatgttctcctccttaactctgcttcgctagcac |
| **O-aroB-R3** | aagaatgaagctggttcattaaacaaaccagatgcgatggtaattgggtagctacgttcgcccag |
| **C-aroB-F** | gcgtgaagttctggaagcgttg |
| **C-aroB-R** | gaccttatcgagatacagaggagcc |
| **O-tktA-F** | gcagcggacgggcgagtagattgcgcaacatgcgagcatgatccagagatgatcgtgcgaacgacctgct |
| **O-tktA-R1** | tggggctccttaatttagcgtggctagcactgtacctaggactgagctagccgtcaacagtagtcgagtcgctccga |
| **O-tktA-R2** | tccatgctcagcgcacgaatagcattggcaagctctttacgtgaggacatgtttggggctccttaatttagcgtgg |
| **C-tktA-F** | gctcattgctaccaataaggcgc |
| **C-tktA-R** | ccacaggacttcggcaatgtca |
| **V-tyrA-F** | GGTACCttgacggctagctcagtcctaggtacagtgctagcccgaagacgctaaggaggtacgagaatggttgctgaattgaccgcattac |
| **V-tyrA-R** | GAGCTCttactggcgattgtcattcgcct |
| **M-tyrA-F1** | cgcgaggcatctatcttggcctcgcgtcg |
| **M-tyrA-R1** | cgacgcgaggccaagatagatgcctcgcg |
| **M-tyrA-F2** | ctggttcggcgattacgtacagcgttttcagagtg |
| **M-tyrA-R2** | cactctgaaaacgctgtacgtaatcgccgaaccag |
| **C-tyrA-F1** | gttccgcttcctttagcagccc |
| **C-tyrA-R1** | gcttccggctcgtatgttgtgtg |
| **D-tyrA-F** | gaaattcatcaggatctgaacgggcagctgacggctcgcgtggcttaagagcatgaccggcgcgatgc |
| **D-tyrA-R** | gatccaacctgatgaaaaggtgccggatgatgtgaatcatccggcactgggctcagcggatctcatgcgc |
| **O-tyrA-F** | gaaattcatcaggatctgaacgggcagctgacggctcgcgtggcttaagaggatccgattttgacggctagctc |
| **O-tyrA-R** | gatccaacctgatgaaaaggtgccggatgatgtgaatcatccggcactgggcctgcaggtcgactctagagg |
| **C-tyrA-F2** | cacgagggcaatcagtcttccg |
| **C-tyrA-R2** | caggccaatcttgaatcagcgg |
| **P-tyrA-F** | ttgacggctagctcagtcctaggtacagtgctagc |
| **P-tyrA-R** | gcatcgcgccggtcatgcttactggcgattgtcattcgcct |
| **P-FKF-F** | aggcgaatgacaatcgccagtaagcatgaccggcgcgatgc |
| **P-FKF-R** | gctcagcggatctcatgcgc |
| **D-tyrR-F** | caggtgaaggttcccatgcgtctggaagtcttttgtgaagaccgactcgggatcgtgcgaacgacctgct |
| **D-tyrR-R** | gaaatcaccaccgagccggattacgaagcagctctggctgtactgaaagccagtagtcgagtcgctccga |
| **C-tyrR-F** | ggtgctgaccggatatctttacgc |
| **C-tyrR-R** | ccctctccactttccgtaacgc |
| **V-ppsA-F** | gcatgaccggcgcgatgc |
| **V-ppsA-R** | ctagctcagtcctaggtacagtgctagcttaactttaatKaggagaNMtacatatgtccaacaatggctcgtcacc |
| **O-ppsA-F** | cgtttataccgatggtttatgtggaaattgtcgaagagagcagatttgcggcatgaccggcgcgatgc |
| **O-ppsA-R** | ccgagttggttataccaaagcaccagcggtgacgagccattgttggacatggtgacgagccattgttggacatatg |
| **C-ppsA-F** | ccgatggtttatgtggaaattgtcg |
| **C-ppsA-R** | cgttttatccagcagttcataaatgcg |
| **O-aroG-F** | gatctcgtttttcgcgacaatctggcgtttttcttgctaattccaggatttgacggctagctcagtcctaggtacagtgctagcgactatttcaaaaggagcatcacgaatgaattat |
| **O-aroG-R** | cggttgcaaaccagggtaaagcgaagtaaacgtcattcgtttaaaatgaggagatgggaattagccatggtcc |
| **V-aroG-F** | aGAGCTCgactatttcaaaaggagcatcacgaatgaattatc |
| **V-aroG-R** | aGGTACCaaagccgactgcaagtgagtcg |
| **M-aroG-F** | caggtgagtttctcaatatgatcacccca |
| **M-aroG-R** | tggggtgatcatattgagaaactcacctg |
| **D-aroG-F** | gatctcgtttttcgcgacaatctggcgtttttcttgctaattccaggatgagatgggaattagccatggtcc |
| **D-aroG-R** | cggttgcaaaccagggtaaagcgaagtaaacgtcattcgtttaaaatgaggtgtaggctggagctgcttc |

^a^ Capital letters indicate restriction sites.

^b^ Underlined sequences indicate homologous sequences.

^c^ Underlined and capital letters indicate randomized sequences for library construction.

**Supplementary Figures and Legends**

**Supplementary Figure S1.** Relationship between predicted expression levels and measured specific enzyme activities of each *ppsA* variant. Predicted expression and activity were highly linearly correlated (R^2^ = 0.69), indicating that the prediction method, UTR Designer, provides exact sequence information for fine-tuning expression levels.


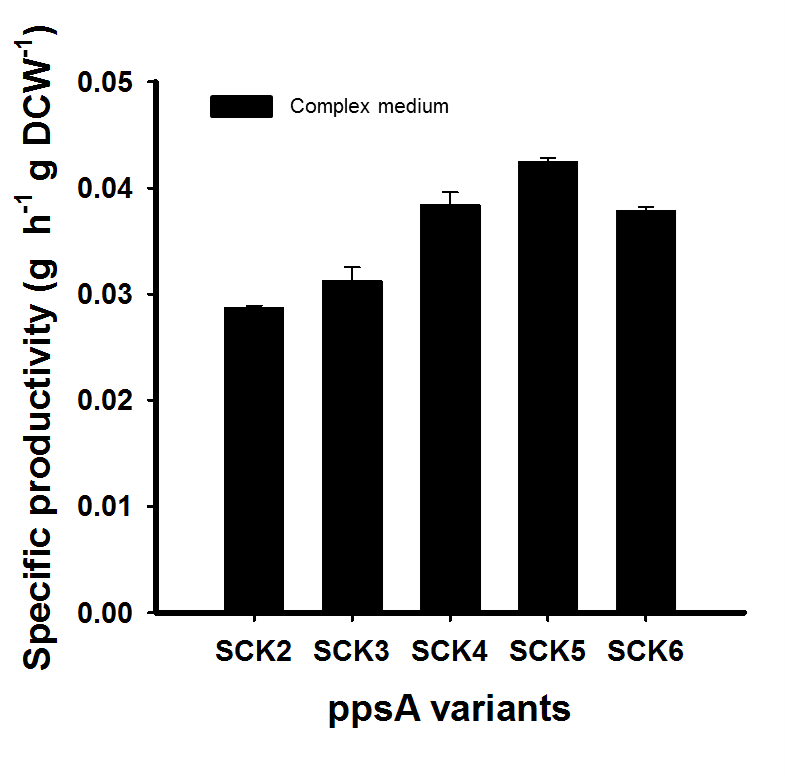


**Supplementary Figure S2.** The specific productivity of L-tyrosine of each ppsA variant after culturing for 16 h in complex medium. The tendency of L-tyrosine productivity was shown like result in M9 minimal medium that the L-tyrosine productivity of P53 was the highest among *ppsA* variants. Each point and error bar indicates means and standard deviations between measurements from biological triplicate cultures.
